# Probiotic Assessment of *Lactobacillus plantarum* 15HN and *Enterococcus mundtii* 50H Isolated from Traditional Dairies Microbiota

**DOI:** 10.15171/apb.2016.07

**Published:** 2016-03-17

**Authors:** Babak Haghshenas, Minoo Haghshenas, Yousef Nami, Ahmad Yari Khosroushahi, Norhafizah Abdullah, Abolfazl Barzegari, Rozita Rosli, Mohammad Saeed Hejazi

**Affiliations:** ^1^ Institute of Biosciences, University Putra Malaysia, 43400 UPM Serdang, Selangor, Malaysia.; ^2^ School of Medicine, Shahid Beheshti University of Medical Sciences, Tehran, Iran.; ^3^ Drug Applied Research Center, Tabriz University of Medical Sciences, Tabriz, Iran.; ^4^ Chemical and Environmental Engineering Department, Faculty of Engineering, University Putra Malaysia, 43400 UPM Serdang, Selangor, Malaysia.; ^5^ Tabriz University of Medical Sciences, Molecular Medicine and Therapy Lab, Research Center for Pharmaceutical Nanotechnology, Tabriz, Iran.; ^6^ Tabriz University of Medical Sciences, Faculty of Pharmacy, Tabriz, Iran.

**Keywords:** Probiotic, Lactic acid bacteria, Adherence, Antimicrobial resistance

## Abstract

***Purpose:*** Probiotics are microorganisms, which show beneficial health effects on hosts once consumed in sufficient amounts. Among probiotic bacteria, the bioactive compounds from lactic acid bacteria (LAB) group can be utilized as preservative agents. LAB group can be isolated and characterized from traditional dairy sources. This study aimed to isolate, identify, and biologically characterize probiotic LAB strains from Iranian traditional dairy products.

***Methods:*** A total of 19 LAB strains were identified by sequencing of their 16S rRNA genes. They were examined for adherence to human intestinal Caco-2 cells and tolerance to low pH/high bile salts and simulated in vitro digestion conditions. Moreover, they were evaluated further to assess their ability to prevent the adhesion of Escherichia coli 026 to the intestinal mucosa, inhibitory functions against pathogens, and sensitivity to conventional antibiotics.

***Results:*** L. plantarum 15HN and E. mundtii 50H strains displayed ≥ 71% survival rates at low pH/high bile salts and ≥ 40% survival rates in digestive conditions. Their adherences to Caco-2 cells were 3.2×105 and 2.6×105 CFU mL-1 respectively and high values of anti-adhesion capability were observed (≥36%). They inhibited the growth of 13 and 11 indicator pathogens respectively. Moreover, they were sensitive or semi-sensitive to seven and three out of eight antibiotics respectively.

***Conclusion:*** L. plantarum 15HN and E. mundtii 50H, which were isolated from shiraz product, displayed above-average results for all of the criteria. Therefore, they can be introduced as novel candidate probiotics that could be used in the food industry.

## Introduction

Probiotics are introduced as microorganisms which show the beneficial effects on the host health when consumed in sufficient amounts.^1^ Among probiotics, LAB group, through secreting the bioactive compounds, can be utilized as preservative agents. Therefore, the majority of probiotics include LAB.^2^ The LAB is a functional heterogeneous bacterial group which is linked to traditional dairy and food fermented products such as yogurt, curd and cheese. *Enterococcus, Leuconostoc, Lactobacillus,* and, *Lactococcus* species are common LAB commonly consumed as probiotics.^3,4^

The health promoting effects of probiotic bacteria are strain-specific, hence, their discrimination and identification is very important and crucial under species level, especially, at strain level. By applying efficient and valid strain-typing techniques, their function can be monitored. Besides that, some typing methods can be utilized for assessment on the safety aspect and ecological properties of probiotics.^5^

LAB exhibit similar growth conditions; as such, these bacteria cannot be accurately identified and differentiated using phenotypic methods such as protein profiling, morphological characterization and carbohydrate fermentation patterns.^6^ There are some disadvantages of identifying and differentiating LAB bacteria by traditional methods which are time consuming, high cost and complicated results.^7^ Hence, to obtain clear classification results, researchers use practical molecular identification methods, such as repetitive sequence-based PCR ((GTG)_5_-PCR),^8^ specific gene sequencing,^9^ 16S rRNA sequencing,^10^ and ribotyping with specific probes.^11^

Many probiotic bacteria are sensitive to low acidic, high bile salt, and enzymatic conditions in the digestive system and are eliminated after consumption.^12^ Meanwhile, a requirement for bacteria to be recognized as a probiotic is their capability to adhere to the intestinal mucosa to avoid being removed from the colon.^13^ Moreover, probiotics can contain antibiotic-resistance genes; thus, high resistance to various antibiotics is induced by transferring these genes to other probiotics/pathogenic bacteria.^14^ Then, these beneficial bacteria should tolerate gastrointestinal environment, adhere to the intestinal mucosa, show high anti-pathogenic activities, and exhibit susceptibility to antibiotics to maximize their health-promoting effects.^15^

Diverse traditional dairy products with health-enhancing benefits, such as improvement of nutrient absorption, inactivation of toxins, and anti-pathogenic activities, are used worldwide.^16^ In different parts of Iran, due to the existence of diverse climate, the wide range of traditional dairy products such as curd, tarkhineh, shiraz, yogurt and cheese are produced. Considering the high acidity of traditional dairy products compared with commercial dairy products and due to limited usage of antibiotics in the rural area of Iran (Kermanshah province), we could possibly obtain bacteria with probiotic properties from these natural resources.

Bacteria were isolated, identified, and clustered from five types of traditional dairy products by using 16S rRNA sequencing. Based on scientific agreements, the characterization and assessment of probiotics properties must be performed by standard *in vitro* experiments.^17,18^ Therefore, this study aimed to screen new strains with high probiotic capability by employing *in vitro* experiments such as low pH (pH 2.5) and bile salt (0.3% oxgall) tolerance tests, survival assay in simulated in vitro digestion conditions, adhesion assay to Caco-2 cells, anti-adhesion assay against *E. coli* (026), antimicrobial assay against 14 human indicator pathogens and susceptibility assay against eight clinically important antibiotics.

## Materials and Methods

### Isolation of bacteria

Between April and July 2012, a total of 100 samples including 20 samples of each fermented dairy products (cheese, yogurt, curd, shiraz, and tarkhineh), were collected in rural area of Kermanshah, Iran. Five g of each dairy sample was suspended in 20 mL sodium citrate solution (pH 7.0) and homogenized using Stomacher 400 Circulator (Seward Laboratory Systems Inc, USA) for 2 min. Then, 1 mL of the samples was added to 10 mL of MRS broth (Merck, Germany) and incubated for 24 h in anaerobic condition (37 ºC, 5% CO_2_). Finally, 0.02 mL of those diluted solutions was spread for 48 h on MRS agar media (Merck, Germany).^19^ The single colonies on the growth agar plate were selected and transferred to 15 mL of broth culture medium and incubated for 24 h at 37 °C. The isolates were stored in 25% (w/v) glycerol at -70 °C for further assessments.

### Amplification and sequencing of 16S rRNA

The amplification of 16S rRNA fragment was conducted in a thermal cycler PTC 200 (MJC research, Waltham, USA) by using a pair of LAB-specific universal primers (Hal_6_F/Hal_6_R) (F: 5’-AGAGTTTGATCMTGGCTCAG-3’ and R: 5’-TACCTTGTTAGGACTTCACC-3’) that have been previously described.^19^ Amplified products were electrophoresed at a constant voltage of 70 V for one h and were visualized in a gel documentation system (Bio-Rad, USA). The DNA fragments with the size of 1500 bp indicate the right amplification. Finally, the amplified 16S rRNA fragments were sequenced by Macrogene (Korea). The sequences were then analyzed using the BLAST program of the National Center for Biotechnology Information (http://www.ncbi.nlm.nih.gov/BLAST), in which the isolated bacteria were identified at strain levels.

### Resistance to acidic condition

A modified method previously described by Conway *et al*. (1987) was used to screen of isolated strains for selecting the resistant ones to the low pH condition. 50 µL respective stock cultures of probiotic candidate were incubated in 5 mL growth medium (MRS broth) at 37 °C for 24 h. Each cell culture media was centrifuged at 2,000 ×*g* for 15 min, the supernatants were removed and the cell plates were re-suspended for 3h in 1 mL PBS (pH 2.5) at 37 °C. By performing the pour plate technique and comparing the results with incubated strains in normal MRS broth (pH 7.4) for 0 and 3 h, the bacterial survival rates were calculated by using the following equation: Survival rate (%) = (log cfu N_1_ /log cfu N_0_) ×100%.

The total viable counts of isolates in MRS agar medium after treatment with the acids are displayed by N_1,_ while N_0_ displays the total viable counts of isolates before incubation in the low pH conditions.^20^

### Survival in bile salts

To determine the resistant bacteria for high bile concentration, a modified method described by Walker & Gilliland, (1993) was used. 50 µL stock-cultures of isolate bacteria were incubated in 5 mL MRS growth medium at 37 °C for 24 h. Then, the respective bacterial cultures (50 µL), similar to aforementioned method, were re-suspended in 5 mL MRS-THIO broth containing MRS supplemented with 0.3% (w/v) oxgall (Sigma-Aldrich, St. Louis, MO, USA, pH 7.0) and 0.2% (w/v) sodium thioglycollate (Sigma-Aldrich, St. Louis, MO, USA) for 3 h at 37 °C. The treated cells were separated on related agar medium and the bacteria maintenance was evaluated using the pour plate technique on MRS agar at time points 0 and 3 h of incubation.^21^ The survival rate for bile resistance was calculated using the following equation: Survival rate (%) = (log cfu N_1_ /log cfu N_0_) ×100%. Total clones after treatment with the bile salts displayed by N_1_ while N_0_ displays the total clones before incubating in bile salt conditions.

### Survival in simulated in vitro digestion 

To assess in vitro digestion, the method previously described by Seiquer *et al.* (2001) was used with some modifications. To recreate the gastric digestion, pepsin with a final concentration of 5% (w/v) was added to the samples, the pH values of which were adjusted to 2.5. The samples were incubated for 120 min at 37 °C with gentle agitation at 110 rpm. To simulate intestinal digestion, the samples were adjusted to pH 6.0, and solutions of bile salts and pancreatin were added at final concentrations of 0.3 and 0.1% (w/v), respectively. The samples were incubated at 37 °C for 180 min with gentle agitation at 110 rpm.^22^ To determine cell count, the samples were removed before and after gastric and intestinal digestion, and the aliquots were serially diluted and plated in triplicate on MRS agar. Then, the plates were incubated for 48 h under anaerobic conditions.

### Adhesion to Caco-2 cells

Bacteria were evaluated for their adhesion capability to the human colon carcinoma cell line Caco-2. The cells were cultured in RPMI medium supplemented with 10% heat-inactivated fetal bovine serum and 1% penicillin–streptomycin mixture. Cells were cultured on 24-well tissue culture plates and incubated at 37 °C in 5% CO_2_ under a relatively humidified atmosphere until a confluent monolayer was formed. Before the adhesion assay, the media in the wells containing a Caco-2 cell monolayer were removed and replaced once with fresh antibiotic-free RPMI. Thereafter, 1×10^7^ cfu/mL of bacteria was added to each well with a total volume of 1 mL and then incubated for 3 h at 37 °C under an atmosphere of 5% (v/v) CO_2_. To remove non-attached bacterial cells, the wells were washed thrice with a sterile pre-warmed PBS solution. To detach the cells from the wells, 1 mL of 1% (v/v) Triton X-100 was added to each well, and the mixture was stirred for 10 min. To measure the viable cell count, the cell suspension was plated onto MRS agar and incubated at 37 °C under anaerobic conditions.^23^ This assay was performed in triplicate.

### Anti-adhesion capability

Competition assay was performed by adding isolated strains and native isolate of *Escherichia coli* (026) simultaneously to the Caco-2 cells in an initial ration of 1:1 followed by incubation for 60 min. Non-attached *E. coli* and LABs were removed by washing thrice with a sterile pre-warmed PBS solution and the bacterial counts were carried using the pour plate technique on MRS agar. Competition rate was calculated as the percentage of adhesive *E. coli* (026) in combination with LAB strains compared to *E. coli*-attached bacteria in the absence of LABs.

In inhibition assay, the isolated strains were added to Caco-2 cells (monolayer) and incubated for 60 min. Then, non-attached LABs were removed by washing and *E. coli* (026) was added to mixture followed by incubation for 60 min. To detach the Caco-2 cells from the wells, 1 mL of 1% (v/v) Triton X-100 was added to each well, and the mixture was stirred for10 min and the bacterial counts were carried using the pour plate technique on MRS agar. The inhibition rate for LABs was calculated using the following equation:

Inhibition rate = 100 × (1 − A1/A2), where the percentage of adhesion by *E. coli* cells in the presence of LABs displayed by A1while A2displays the percentage of adhesion by *E. coli* cells in absence of LAB strains.

In displacement assay,* E. coli* (026) were added to Caco-2 cells (monolayer) and incubated for 1 h. Then, non-attached *E. coli* (026) cells were removed and LAB strains were added to the Caco-2 cells followed by incubation for 1 h. Caco-2 and bacterial cells were detached from the wells and the bacterial counts were carried using the pour plate technique on MRS agar. Displacement rate for LAB strains was calculated as the percentage of adhesive *E. coli* (026) in combination with LAB strains compared to *E. coli* -attached bacteria in the absence of LAB strains.^24^ These three assays were performed in triplicate.

### Antimicrobial activity

The modified agar diffusion well method previously described by Bauer *et al.* (1966) was used to determine the antibacterial activities of isolated bacteria.

The overnight cultured isolated strains in MRS broth medium at 37 °C were filtered through 0.2 µm filter, and then 50 µL of each filtrate was added to 7 mm diameter wells on Mueller-Hinton agar plates (Sigma-Aldrich, USA), which before were incubated overnight by indicator pathogens at 37 °C. In certain cases, the isolated active supernatants had low pH. Thus, the pH of the isolated active supernatants was adjusted by adding NaOH to the physiological solution (pH 7.2) for use in the antimicrobial assay experiments. After overnight incubation of plates at 37 °C, the clear zones around of each well were measured and considered as positive antibacterial activity.^25^ According to diameter of inhibition zone; the anti-pathogen activity was divided to strong (≥ 20 mm), moderate (20 mm ≥ diameter ≥ 10 mm), and weak (≤ 10 mm).^26^ The means data of experiment for twice with three repeats in each time were calculated and considered for each bacterial isolates.

### Antibiotic susceptibility 

The modified disc diffusion method by using some clinically important antibiotics such as chloramphenicol, vancomycin, tetracycline, erythromycin, ampicillin, gentamycin, clindamycin, and penicillin was performed to determine the antibiotic susceptibility of each isolated strain. The LAB group in anaerobic condition was incubated for 24 h in MRS broth medium at 37 °C. Then, 50 μL of the diluted cultures (approximate 10^6^-10^7^ viable cells) were diffused onto Mueller-Hinton agar. After spreading of each strain on Mueller-Hinton agar plates, the antibiotic disks purchased from PadtanTeb Co (Tehran, Iran), were manually placed on plates by using sterilized forceps. These plates were subjected to incubation for 24 h at 37 °C, and after incubation time, the clear zones around of each disc were measured.^27^ Based on the areola diameters and antibiotic discs producer’s guidelines, and also according to recommended standards (Performance Standards for Antimicrobial susceptibility testing, from Clinical and Laboratory Standards Institute, Wayne, PA, CLSI 2007), the strains were grouped to sensitive, intermediate and resistant at viewpoint of antibiotic susceptibility.^28^ The antibiotic susceptibility assay was performed in triplicate.

## Results and Discussion

### Isolation and identification of LAB strains

The presence of LAB strains in the isolated samples was confirmed by amplifying the 16S rRNA genes. In total, 19 strains were identified as LAB. These strains belonged to four genera (*Lactococcus*, *Leuconostoc*,* Lactobacillus*, and *Enterococcus*).

More than 70% of isolates were LAB which was isolated from all these five dairy products. *Leuconostoc* with more than 42% and *Lactobacillus* with less than 11% had the maximum and minimum LAB populations. According to results, four strains belonged to four genera (*Lactococcus*, *Leuconostoc*, *Lactobacillus*, and *Enterococcus*) were isolated from yogurt. In curd, four strains belonged to three genera (*Leuconostoc*, *Lactobacillus*, and *Enterococcus*) were observed while in cheese, five strains belonged to *Lactococcus* and *Leuconostoc* genera were identified. Meanwhile, shiraz had 4 strains belonged to *Enterococcus*, *Lactobacillus*, and *Leuconostoc* genera while in tarkhineh, 2 strains (*Leuconostoc*) were observed (Table 1).

**Table 1:** Isolated LAB strains and their survival rates after 3 h incubation at pH 2.5 and 0.3% bile salts

**Strain code**	**Species**	**Origin**	**Low pH survival (%)**	**Bile salt survival (%)**
46Lac	*Lactobacillus paracasei subsp. paracasei*	yogurt	76	92
15HN	*Lactobacillus plantarum*	shiraz	71	88
41Lac	*Leuconostoc mesenteroides subsp. mesenteroides*	curd	84	90
18H	*Leuconostoc mesenteroides subsp. mesenteroides*	tarkhineh	53	74
35C	*Leuconostoc mesenteroides subsp. mesenteroides*	tarkhineh	47	70
2H2	*Leuconostoc mesenteroides subsp. mesenteroides*	cheese	61	80
10H2	*Leuconostoc mesenteroides subsp. cremoris*	yogurt	43	65
19H2	*Leuconostoc mesenteroides subsp. cremoris*	cheese	55	77
5H	*Leuconostoc lactis*	cheese	49	66
15H	*Leuconostoc lactis*	shiraz	64	82
44Lac	*Lactococcus lactis subsp. lactis*	cheese	85	94
13H2	*Lactococcus lactis subsp. lactis*	curd	53	71
13H	*Lactococcus lactis subsp. lactis*	curd	66	82
44L	*Lactococcus lactis subsp. cremoris*	cheese	81	95
11H	*Lactococcus lactis subsp. cremoris*	yogurt	44	65
13C	*Enterococcus faecalis*	curd	73	98
50H	*Enterococcus mundtii*	shiraz	78	98
50H2	*Enterococcus mundtii*	shiraz	51	70
39C	*Enterococcus durans*	yogurt	82	96
1058	*Lactobacillus. plantarum*	PTCC	63	83

On the basis of FAO/WHO guidelines and our results, analyzing and identifying probiotic microorganisms with 16S rRNA sequencing patterns can be considered as an accessible, cost-effective, and suitable technique compared with other costly and time-consuming molecular techniques.^18^ This technique has also been utilized as an effective method to analyze and isolate different LAB genera which were isolated from fermented dairy products.^29,30^

The biodiversity of LAB species in fermented dairy products is variable and region specific. According to our findings, *L. plantarum* and* L. paracasei* subsp. *paracasei* were isolated from traditional Iranian dairy products. But, in traditional Spanish cheese (Armada cheese); the predominant *Lactobacilli* are *L. casei ssp. casei* and *L. brevis.*^31^ Meanwhile, in Greek goat cheese (Batzos cheese), *L. paracasei ssp.* and* L. sake* are the dominant species,^32^ while in Brazilian fresh cheese (Minas Frescal cheese) the predominant species is *L. acidophilus*.^33^ It revealed that, our results are quite different from those in terms of species and prevalence.

### Resistance to acidic condition

Many probiotic bacteria are delivered to the body through foods; therefore, probiotics should tolerate low pH levels in the stomach and survive for a minimum of 90 min before they can colonize the gastrointestinal tract and elicit health-promoting effects.^34^ Based on the results, all selected LAB strains retained their viability even after 3 h of exposure to pH 2.5 (Table 1). The survival rates ranging from 71 to 76% were observed in *Lactobacillus* strains, whereas the survival rates of *Lactococcus* and *Leuconostoc* strains ranged from 43 to 85%. Moreover, *Enterococcus* strains showed tolerance ranging from 51 to 82% to acidic conditions.

*Enterococcus* strains showed better low pH tolerance than others. This high tolerance capability can be related to the bilayer membrane structure, which enables easy tolerance of inverse conditions.^35^ There are limited information about the acidic tolerance of this genus, but the high survival rates similar to our results was observed for some species such as *E. faecium* and *E. faecalis* after incubation at pH 3.0.^36^ The LAB strains with the most efficient tolerance to acidic conditions were *L. lactis* subsp*. lactis* 44Lac*, L. mesenteroides* subsp*. mesenteroides* 41Lac*, E. durans* 39C and *L. lactis* subsp*. cremoris* 44L with survival rates of 85, 84, 82 and 81%, respectively.

### Survival in bile salts

Probiotics should also survive bile salt defense mechanisms.^12^* In vitro* bile tolerance experiments show the same cell viability results. Then, the tolerance of probiotic bacteria to gastrointestinal conditions can be assessed by *in vitro* methodology at the same oxgall concentration [0.3% (w/v)].^35^

All 19 isolated strains displayed high tolerance to bile salt conditions, ranging from 6 to 25% higher than their low pH tolerance. The survival rates of *Lactobacillus* strains ranged from 88 to 92%, whereas the survival rates of *Lactococcus* and *Leuconostoc* strains ranged from 65 to 95%. Same as acidic condition, the best survival rates among LAB were observed for *Enterococcus* strains which ranged from70 to 98% (Table 1). The same results showed that among LAB group, *Enterococcus* species, especially *E. faecium,* had the highest tolerance capability in the bile salts conditions.^37^ The strains with the highest tolerance to 0.3% oxgall were *E. mundtii* 50H, *E. faecalis* 13C and *E. durans* 39C with the survival rates of 98, 98 and 96%, respectively. The effects of bile salts on bacterial cells are different from acidic effects, but combined results were obtained in this study. Stress adaptation mechanisms triggered by acidic environments can result in bile salt resistance which can be unpredictable and higher than the resistance to acidic conditions.^12^

*L. paracasei* subsp*. paracasei* 46Lac, *L. plantarum* 15HN, *L. mesenteroides* subsp*. mesenteroides* 41Lac, *L. lactis* subsp*. lactis* 44Lac, *L. lactis* subsp*. cremoris* 44L, *E. faecalis* 13C, *E. mundtii* 50H, and *E. durans* 39C showed high survival rates under low pH (>70%) and high bile conditions (>87%). Consequently, these eight strains were selected for further probiotic analysis.

### Survival in simulated in vitro digestion

A requirement for bacteria to be recognized as a probiotic is their capability to remain alive while passing through the upper digestive tract to reach the large intestine, where their useful actions are expected.^38^

A total of eight isolated LAB (most resistant strains to low pH and bile salts conditions) were tested to evaluate the strains further through a simulated digestion test. Among these isolates, only five strains survived exposure to the simulated digestion conditions of the stomach.

The resistant strains were *L. plantarum* 15HN,* L. lactis* subsp. *cremoris* 44L, *E. faecalis* 13C, *E. mundtii *50H, and *E. durans* 39C. These resistant strains were isolated from shiraz, cheese, curd, shiraz, and yogurt, respectively. The highest percentage of survivability was observed for *L. plantarum* 15HN and *E. mundtii *50H, with survivability values of 42 and 40%, respectively (Table 2). Similar to our results, high survival rates was reported for three commercial probiotic strains including *L. casei* subsp. *shirota, L. casei* subsp. *immunitas,* and* L. acidophilus* subsp. *Johnsonii* in simulated digestion condition.^39^ Based on our results, the resistance to simulated *in vitro* conditions is strain-specific because of a wide range of diversity in survivability even among the same species.

**Table 2:** The survival rates of low pH (pH 2.5) and bile salt (0.3 % (w/v) oxgall) resistant strains in simulated digestion conditions and their capacity to adhere to the Caco-2 cell line

**Isolated Strains**	**Digestion** **Survival (%)**	**Adhesion to Caco-2 no.** **of cells (CFU mL**^-1^)
*L. paracasei*subsp.*paracasei*46Lac	0	-
*L. plantarum*15HN	42	3.2×10^5^
*L. mesenteroides* subsp. *mesenteroides* 41Lac	0	-
*L. lactis* subsp. *Lactis* 44Lac	0	-
*L. lactis* subsp. *cremoris* 44L	31	2.8×10^3^
*E. faecalis* 13C	38	4.8×10^4^
*E. mundtii *50H	40	2.6×10^5^
*E. durans* 39C	28	2.2×10^3^

-: Not determined

### Adhesion to Caco-2 cells

One of the most desirable features of probiotics is their capability to remain alive in the gastrointestinal tract. To be colonized in the intestine, probiotic bacteria have to adhere to the intestinal mucosa to avoid being removed from the colon by peristalsis.

These five digestion-resistant LAB strains were examined for their capability to adhere to Caco-2 cells. The results showed that *L. plantarum* 15HN and *E. mundtii *50H were the most adherent strains, with adhesion values of 3.2×10^5^ and 2.6×10^5^ CFU/mL, respectively (3.2 and 2.6% of adhesion, respectively) (Table 2). Interestingly, these isolates were the most resistant strains to digestion conditions. These results are in accordance with that of other studies, which showed that some *L. plantarum* strains, such as *L. plantarum* L2,* L. plantarum* CH3 and CH41, could adhere to Caco-2 cells better than other *Lactobacilli* (1.6 – 1.8% of adhesion)*.*^40,41^ Similar to our results, high capability to adhere to Caco-2 cells was reported for *Enterococcus strains*.^42,43^

### Anti-adhesion capability

To evaluate the ability of isolated strains to prevent the adhesion of pathogens to the intestinal mucosa, different anti-adhesion assays including competition, inhibition, and displacement assays were carried out. Among the isolated LAB, only two strains survived exposure to the simulated digestion conditions of the stomach and showed high capability to adhere to Caco-2 cells. Then, for further probiotic assessments, the ability of *L. plantarum* 15HN, *E. mundtii *50H and the probiotic control *L. plantarum* PTCC 1058 was evaluated to compete, inhibit and displace the attachment of *E. coli* (026) to Caco-2 cell lines.

**Figure 1:**
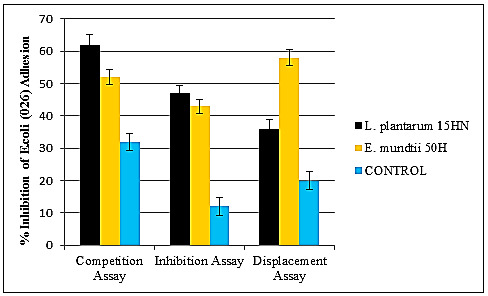
Anti-adhesion assays (competition, inhibition and displacement) of *E. coli* (026) in presence of *E. mundtii* 50H, *L. plantarum*15HN and *L. plantarum* PTCC 1058. Results are mean ± standard error. PTCC: Persian Type Culture Collection (Tehran, Iran).

When *E. coli* (026) and LAB strains were added simultaneously (competition assay), *E. coli* had an adhesion of 5.74%. The results showed that degree of adhesion of *E. coli* was reduced by around 32 - 62% (Figure 1). The highest decrease in adhesion of *E. coli* was observed for *L. plantarum*15HN with decreased values of 62%. These results are in accordance with that of other studies, which showed the attachment inhibition of *E. coli* to Caco-2 cells by some LAB strains, such as *L. plantarum* and *L. rhamnosus.*^24,44^

In inhibition assay, *L. plantarum* 15HN and *E. mundtii *50H were the most effective strains which inhibiting the adhesion of *E. coli* (43 - 47%), while *L. plantarum* PTCC 1058 (probiotic control) showed the lowest values of inhibition with 12% (Figure 1). The high values of inhibition observed by *L. plantarum* 15HN and *E. mundtii *50H (>43%) could be due to high competition of these strains with *E. coli (026)* for common adhesion receptors.^45^

In displacement test, same as competition and inhibition assays, *L. plantarum* 15HN and *E. mundtii *50H were the most effective strains which displace with the adherent *E. coli* (36 - 58%) (Figure 1). This high displacement capability could be due to competition for the common adhesion receptors, production of anti-adhesion compounds, and secretion of antimicrobial factors.^46^

The results of the anti-adhesion assays were different from each other. It appears to confirm that different mechanisms are implied in bacterial anti-adhesion processes. The similar observations were documented by other researches.^44,47^ Meanwhile, correlation between the anti-adhesion and adhesion results could suggest that the similar mechanisms implied in both phenomena. By contrast, the absence of any correlation between the anti-adhesion and adhesion results was reported by other researches.^47,48^

### Antimicrobial activity

The most important health-promoting properties of probiotics are their inhibitory functions against pathogens.^49^ Antibacterial activity assessments were conducted against clinically important human pathogens listed in Table 3. In this test, the antagonistic activity of isolated strains and the probiotic control (*L. plantarum* PTCC 1058) against fungi, Gram-positive, and Gram-negative pathogenic bacteria were assessed by formation of inhibition zones. Both two isolated strains, including *L. plantarum* 15HN and *E. mundtii *50H displayed significant anti-pathogenic activities against indicator microorganisms (Table 3).

*L. plantarum* 15HN showed the most efficient antagonistic activity and inhibited the growth of 13 indicator pathogens. Meanwhile*, E. mundtii* 50H exhibited an overall good antagonistic activity and inhibited the growth of most indicator pathogens (Table 3). Similar to this finding, the high antagonistic activities for *L. plantarum*^50^ and *E. mundtii*^51^ against the high diversity of pathogenic bacteria and fungi were reported. Our findings were supported by evidence showing the high anti-microbial activities of *Lactobacillus* and* Enterococcus* strains on diverse pathogens due to secretion of anti-pathogenic proteins (bacteriocin), bio-surfactants, hydrogen peroxide (H_2_O_2_) and organic acids.^52^

Both strains had inhibition activities on *S. flexneri, P. aeruginosa* and *K. pneumoniae*. This sensitivity may be related to the thin cell walls and susceptibility of these Gram-negative bacteria to acidic metabolites. *S. flexneri* and *P. aeruginosa* are involved in different hospital-acquired infections, such as wound and urinary tract infections; hospital outbreaks caused by their antibiotic-resistant strains have also been reported.^53,54^ Meanwhile, *K. pneumonia* causes different diseases, such as pneumonia and urinary tract infections, particularly among the elderly with weakened immune system; *K. pneumoniae* strains also show high rates of resistance to antibiotics.^55^ Therefore, these isolated LAB from safe sources and with high antagonistic activities may be prescribed against antibiotic-resistant strains of these two Gram-negative bacteria, particularly for the treatment of individuals with weak immune system.

**Table 3:** The inhibitory effect of isolated strains against pathogenic microorganisms

**Pathogens**	***L. plantarum*** **15HN**	***E. *** ***mundtii*** *** *** **50H**	***L. *** ***plantarum*** ** PTCC 1058**
*Pseudomonas aeruginosa (PTCC 1181)*	11.3±0.3	16.7±0.3	10.0±0.0
*Candida albicans (PTCC 5027)*	10.0±0.0	13.0±0.0	0.0±0.0
*Serratia marcesens (PTCC 1187)*	17.3±0.0	14.0±1.0	0.0±0.0
*Enterococcus faecalis (PTCC 1394)*	0.0±0.0	12.3±1.2	0.0±0.0
*Staphylococcus saprophyticus (PTCC 1440)*	11.3±0.7	0.0±0.0	0.0±0.0
*Streptococcus mutans (PTCC 1683)*	17.3±0.6	0.0±0.0	0.0±0.0
*Escherichia coli (PTCC 1276)*	10.0±0.0	13.3±0.3	0.0±0.0
*Salmonella typhimurium (ATCC 14028)*	12.3±1.2	13.3±0.3	0.0±0.0
*Staphylococcus aureus (ATCC 25923)*	11.7±0.3	13.0±0.0	0.0±0.0
*Escherichia coli (026)*	12.3±0.7	15.7±0.3	0.0±0.0
*Bacillus cereus subsp. kenyae (PTCC 1539)*	10.0±0.0	0.0±0.0	0.0±0.0
*Listeria monocytogenes (PTCC 1163)*	13.7±0.9	17.0±0.0	0.0±0.0
*Klebsiella pneumoniae (PTCC 1053)*	12.0±0.6	13.3±1.2	11.7±0.9
*Shigella flexneri (PTCC 1234)*	12.0±0.0	14.0±0.6	12.3±0.7

Values are the mean ± standard deviations of triplicate measurements; the data represent the mean diameters (mm) of areola around replicated wells.

### Antibiotic susceptibility 

The sensitivity of probiotics to conventional antibiotics is a fundamental health promoting characteristic. Overuse of antibiotics can spread the resistance genes across a region and transfer to other microorganisms societies.^14^ The antibiotic susceptibility patterns were strain specific. *L. plantarum* 15HN displayed the best results and were sensitive or semi-sensitive to seven out of eight antibiotics. This strain just was resistant to chloramphenicol. On the other hand, *L. plantarum* PTCC 1058 (probiotic control) by resistance to six antibiotics (erythromycin, ampicillin, vancomycin, chloramphenicol, clindamycin and penicillin), was the most resistant strains (Table 4). Vancomycin is one of the last antibiotics which is highly effective against clinical infections caused by multidrug-resistant pathogens then resistance against vancomycin is critical among probiotics.^56,57^

**Table 4:** Antibiotic susceptibility of isolated strains against the high consumption antibiotics

**Isolated Strains**	**Diameter of inhibition zone (mm)**							
	**C**	**TE**	**ER**	**AM**	**GE**	**CC**	**P**	**V**
*L. plantarum*15HN	R	S	I	S	S	S	S	S
*E. mundtii *50H	R	S	R	R	S	S	R	R
*L. plantarum* PTCC 1058	R	S	R	R	S	R	R	R

C: chloramphenicol; TE: tetracycline; ER: erythromycin; AM: Ampicillin; GE: gentamycin; CC: clindamycin; P: penicillin; V: vancomycin

**Figure 2:**
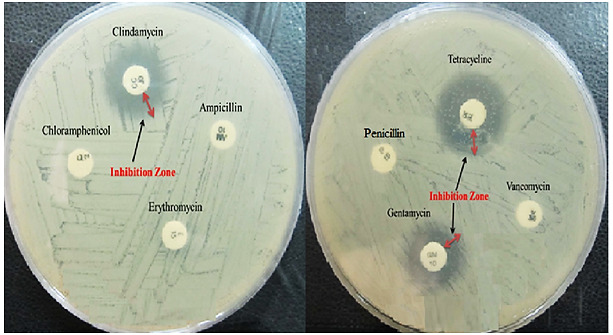
The susceptibility spectrum of *E. mundtii* 50H strain against eight antibiotics.

E. mundtii 50H was resistant to erythromycin, ampicillin, vancomycin, chloramphenicol, and penicillin (Figure 2). The recent studies displayed the same resistance capability for the food product’s Enterococci.^58,59^ According to various reports, different strains of this genus such as L. casei, L. salivarius, L. plantarum, L. leishmannii and L. acidophilus carry the vancomycin resistance genes which recommend our results.^60,61^ The recent studies displayed the same resistance capability for the food product’s Enterococci.^58,59^ This genus must be carefully prescribed as probiotic, because similar to our result, an increased growth of Antibiotic-Resistant Enterococci (ARE) such as Vancomycin-Resistant Enterococci (VRE) was observed among some species such as E. faecalis. They can transfer the antibiotic resistance genes to other pathogens such as methicillin-resistant Staphylococcus aureus (MRSA) and make a dilemma in treatment of patients.^61,62^

Both two isolated LAB were sensitive or semi-sensitive to gentamycin, clindamycin and tetracycline. Hence, these antibiotics can be used in their selective growth media but re-establishment of probiotic balance in the gut tract must be considered after antibiotic treatment. In contrast to our results, the high resistance to gentamycin, clindamycin and tetracycline among the probiotic bacteria was reported by other researches.^63,64^

In conclusion, traditional dairy products in west Iran were preliminary screened because different preparation and processing methods can be possibly used as a valuable tool to introduce novel and promising probiotic bacteria. Our findings indicated that *L. plantarum* 15HN and *E. mundtii *50H strains, which were isolated from shiraz, successfully survived in digestive conditions, displayed an acceptable adherence to Caco-2 cells, showed a desirable anti-adhesion capability against *E. coli,* and had a favorable anti-pathogen activity and antibiotic susceptibility. Hence, these two strains have good potential to be used as probiotics.

## Acknowledgments

The financial support of the University Putra Malaysia and the Faculty of Pharmacy, Tabriz University of Medical Sciences, Tabriz, Iran are gratefully acknowledged.

## Ethical Issues

No ethical issues were promulgated.

## Conflict of Interest

The authors declare no conflict of interests. No writing assistance was utilized in the production of this manuscript.
